# 2302 Impacts of postnatal nest change on early development

**DOI:** 10.1017/cts.2018.85

**Published:** 2018-11-21

**Authors:** Antonia P. Francis, Anna Kuznetsova, Keith Martinez, Maria Gloria Dominguez-Bello

**Affiliations:** 1 H+H Clinical and Translational Science Institute, NYU; 2 New York University Undergraduate, New York, NY, USA; 3 Department of Medicine, New York University Langone Medical Center, New York, NY, USA; 4 Department of Microbiology, New York University Langone Medical Center, New York, NY, USA; 5 Sackler Institute, New York University School of Medicine, New York, NY, USA; 6 Department of Biochemistry and Microbiology, and of Anthropology, Rutgers University, NJ, USA

## Abstract

OBJECTIVES/SPECIFIC AIMS: It has been reported that birth mode affects development, with cesarean section born mice gaining more body weight during development. Since mice C-sections involve fostering and nest change, we sought to determine whether changing only the nest and cage would have an effect on development. METHODS/STUDY POPULATION: A total of 53 mice were born to 9 dams, and 21 babies (4 litters) were exchanged in pairs to foreign cages and nests. Litters were followed for body weight and mothers were observed during periods for maternal and nonmaternal behaviors. RESULTS/ANTICIPATED RESULTS: The results show that average body weight was significantly higher in the experimental group in both genders, with 20% higher body weights at weaning. The mothers from the litters that were changed to a new nest showed significantly more non-maternal behavior in the first 2 days if life, than the controls. DISCUSSION/SIGNIFICANCE OF IMPACT: The results suggest that changes in maternal behavior may be linked to the increased weight gain in their babies.

